# Supplementary material for: Dietary Glycemic Index and Glycemic Load Are Not Associated with the Metabolic Syndrome in Lebanese Healthy Adults: A Cross-Sectional Study
Source: Nutrients. 2020 May 13;12(5):1394. doi: 10.3390/nu12051394 (PMC7284586; doi:10.3390/nu12051394)
Supplement: Supplementary file 1 [file nutrients-12-01394-s001.pdf]

Table S1 GI and GL values of food items in the FFQ

Food item	GI 1[a]	GI 2[b]	Serving(g)	GL 1[a]/svg	GL 2[b]/svg
**Bread, white**	95	95	30	15	15
**Bread, brown**	68	68	30	9	9
**Traditional (markouk/tannour)**	97	97	30	15	15
**Breakfast cereals (regular, sugar coated, chocolate, bran)**	66.6	66.6	30	16	16
**Kaak**	81	81	25	15	15
**Rice, white, cooked**	64	64	150	23	23
**Pasta/noodles, plain, cooked**	45.5	45.5	180	20	20
**Wheat/bulgur, cooked**	48	48	150	12	12
**Whole grain rice/pasta/cereals**	37	37	180	16	16
**Milk, skim/low-fat (0-2%)**	32	32	250	4	4
**Milk, whole-fat**	27	27	250	3	3
**Yogurt, fat-free/low fat**	27	27	200	7	7
**Yogurt, whole-fat**	36	36	200	3	3
**Cheese, regular/yellow**	x	27	250	x	3
**Cheese, low fat, white**	x	32	250	x	4
**Labneh, regular**	36	36	200	3	3
**Labneh, low-fat**	27	27	200	7	7
**Citrus orange/ grapefruit**	33.5	33.5	120	4	4
**Peach, plum, prunes**	40.5	40.5	120	5	5
**Strawberries**	40	40	120	1	1
**Grapes**	46	46	120	8	8
**Banana, apples**	45	45	120	9	9
**Dried fruits**	66	66	60	26	26
**Fruit juice, fresh**	45	45	250	13	13
**Fruit juice canned/bottle**	66	66	250	13	13
**Fruits, canned**	50.5	50.5	120	7	7
**Salad green**	x	32	138	x	0.5
**Dark green or deep yellow**	x	37	138	x	1.9
**Tomatoes, fresh**	x	38	123	x	1.2
**Corn/green peas, fresh**	51	51	80	6	6
**Corn/green peas, canned**	46	46	80	7	7
**Potatoes, baked/boiled/mashed**	50	50	150	14	14
**Zucchini/eggplants cooked**	28.3	44.3	65	1.9	2.5
**Cauliflower, cabbage, broccoli**	x	32	123	x	1
**Other canned vegetables (mushroom, palmetto, asparagus)**	x	32	123	x	3.3
**Vegetable juice, fresh**	40	40	250	7	7
**Legumes: Lentils, beans, chickpeas dried, cooked**	28	28	150	7	7
**Legumes canned**	52	52	150	9	9
**Nuts and seeds**	18	18	55	2	2
**Red meat**	x	x	x	x	x

**Poultry**	x	x	x	x	x
**Fish/seafood**	x	50	100	x	4
**Fish (canned)**	x	50	x	x	x
**Eggs**	x	50	44	x	0.3
**Organ meats**	x	50	100	x	3.2
**Luncheon meats**	x	50	30	x	0.3
**Sausages, uncanned**	28	28	100	1	1
**Sausages, hotdogs, canned**	x	28	100	x	0.1
**Veg oil, corn/sunflower/soya**	x	x	x	x	x
**Olive Oil (including with thyme)**	x	x	x	x	x
**Olives**	x	50	22	x	0.3
**Butter**	x	50	15	x	0.0045
**Ghee**	x	50	15	x	0.06
**Mayonnaise**	x	50	13.8	x	0.04
**Tahini**	x	x	x	x	x
**Cakes/cookies/donuts/muffins/croissants/biscuits**	66	66	51	16	16
**Ice cream**	61	61	50	8	8
**Chocolate bar**	50	50	60	18	18
**Sugar, honey, jam, choc**	44	44	22	7	7
**Arabic sweets (baklava, maamoul, knefe)**	59	59	x	x	21
**Soft drink**	63	63	250	16	16
**Soft drink, diet**	x	x	x	x	x
**Turkish coffee**	x	x	x	x	x
**Instant coffee/ tea**	x	50	240	x	0.4
**Cocoa / hot chocolate**	51	51	250	12	12
**Beer**	66	66	250	5	5
**Wine**	x	61	104	x	1.65
**Liquor, whiskey, vodka, rum**	x	61	45	x	x
**Water**	x	x	x	x	x
**Manaeesh, zaatar/cheese**	36	36	100	9	9
**French fries**	75	75	150	22	22
**Potato chips/tortilla**	57	57	150	26.5	26.5
**Falafel, without bread**	15.6	29.5	27	0.5	0.9
**Shawarma**	70	70	x	x	23.8
**Burgers**	66	66	95	17	17
**Pizza**	36	36	100	9	9
**Canned/pre-packed soups**	58	58	250	11	11
**Ketchup**	x	60	15	x	2.4
**Mustard**	x	x	x	x	x

Abbreviations: GI: Glycemic index; GL: Glycemic load; FFQ: Food frequency questionnaire; svg: Serving.

[a] Values based on Approach 1(International table): considering only carbohydrate-rich foods [35].

[b] Values based on Approach 2: same as Approach 1 in addition to GI and GL values proposed by studies (Schulz et al., 2005; van Bakel et al., 2009) [44,46] and USDA CSFII 94-96 food codes [47] with the help of NutritionistPro records at the American University of Beirut (AUB).

Table S2 Energy and macronutrient intakes of study participants across quartiles of GI (1)

	Q1	Q2	Q3	Q4	Significance
**Energy (kcal/d)**	2772.8	3238.4	3322.5	3186.8	p= 0.056
**Carbohydrates (g/d)**	332.4	397	400	420.5	**p= 0.006**
**% Kcal carbohydrates**	48.5	49.7	49.5	53.6	**p= 0.001**
**Total sugar (g/d)**	109.4	114.8	115	80.3	**p= 0.001**
**% Kcal total sugar**	16.3	14.1	14.4	10.4	**p= 0.000**
**Protein (g/d)**	88.6	103	111	108	p= 0.125
**% Kcal Protein**	13	12.7	13	13.3	p= 0.876
**Total Fat (g/d)**	124.5	140.5	144.1	117.6	**p= 0.042**
**% Kcal total fat**	41.9	40.5	39.6	34.6	**p= 0.000**
**SFA (g/d)**	36.8	39.4	40.1	32	p= 0.073
**% Kcal SFA**	11.4	10.8	10.2	9	**p= 0.000**
**MUFA (g/d)**	45.7	51.4	53.5	44.3	p= 2.135
**% Kcal MUFA**	14.8	14.1	14.3	12.3	p= 5.272
**PUFA (g/d)**	32	38.1	39	31.4	p= 2.862
**% Kcal PUFA**	10.5	10.5	10.5	9	p= 2.641
**Cholesterol (mg/d)**	291.7	310.6	386	273.5	p= 2.901
**Fiber (g/d)**	28	29.7	27.7	26.8	p= 0.531

Abbreviations: GI: Glycemic index; Q: Quartile; SFA: Saturated Fatty Acids; MUFA: Monounsaturated Fatty Acids; PUFA: Polyunsaturated Fatty Acids.

Numbers in bold are statistically significant (p-value ≤ 0.05).

Table S3 Total dietary GI and GL intake of participants with and without MetS

	**Participants without MetS (n=181)**	**Participants with MetS (n=102)**	**Significance**
**Glycemic Index 1**			0.053
Q1	49 (27.1)	20 (19.6)	
Q2	48 (26.5)	24 (23.5)	
Q3	47 (26)	24 (23.5)	
Q4	37 (20.4)	34 (33.3)	
**Glycemic Index 2**			0.076
Q1	49 (27.1)	20 (19.6)	
Q2	49 (27.1)	23 (22.5)	
Q3	46 (25.4)	24 (24.5)	
Q4	37 (20.4)	34 (33.3)	
**Glycemic Load 1**			0.050
Q1	49 (27.1)	21 (20.6)	
Q2	44 (51.4)	27 (26.5)	
Q3	50 (27.6)	22 (21.6)	
Q4	38 (21)	32 (31.4)	
**Glycemic Load 2**			0.058
Q1	49 (27.1)	21 (20.6)	
Q2	44 (24.3)	27 (26.5)	
Q3	49 (27.1)	22 (21.6)	
Q4	39 (21.5)	32 (31.4)	

Abbreviations: GI: Glycemic index; GL: Glycemic load; MetS: Metabolic syndrome; Q: quartile.

Table S4 Multivariable logistic regression analyses of MetS components and adiposity indicators by dietary GI 1 quartiles

	Daily Glycemic Index 1			
	**Quartile 1** (n=71)	**Quartile 2** (n=72)	**Quartile 3** (n=72)	**Quartile 4** (n=71)
	OR (95% CI)			
**Elevated triglycerides**				
Crude model	1	1.338 (0.617-2.903)	1.364 (0.628-2.961)	**2.157 (1.022-4.552)**
Model 1[a]	1	1.193 (0.539-2.642)	1.251 (0.565-2.769)	1.788 (0.827-3.867)
Model 2[b]	1	1.340 (0.582-3.086)	1.297 (0.564-2.986)	1.672 (0.739-3.783)
Model 3[c]	1	1.340 (0.582-3.086)	1.297 (0.564-2.986)	1.672 (0.739-3.783)
**Elevated fasting blood glucose**				
Crude model	1	**0.464 (0.225-0.957)**	0.673 (0.336-1.348)	1.098 (0.561-2.147)
Model 1[a]	1	**0.377 (0.175-0.810)**	0.572 (0.276-1.185)	0.655 (0.312-1.373)
Model 2[b]	1	**0.380 (0.174-0.833)**	0.550 (0.260-1.167)	0.598 (0.277-1.288)
Model 3[c]	1	**0.380 (0.174-0.833)**	0.550 (0.260-1.167)	0.598 (0.277-1.288)
**Elevated blood pressure**				
Crude model	1	1.721 (0.808-3.665)	1.222 (0.560-2.670)	1.757 (0.824-3.745)
Model 1[a]	1	1.517 (0.672-3.423)	1.047 (0.452-2.421)	1.014 (0.437-2.351)
Model 2[b]	1	1.560 (0.659-3.690)	0.938 (0.387-2.272)	0.803 (0.328-1.961)
Model 3[c]	1	1.560 (0.659-3.690)	0.938 (0.387-2.272)	0.803 (0.328-1.961)
**Reduced HDL-C**				
Crude model	1	0.868 (0.441-1.708)	0.887 (0.450-1.748)	1 (0.510-1.960)
Model 1[a]	1	0.868 (0.441-1.708)	0.887 (0.450-1.748)	1 (0.510-1.960)
Model 2[b]	1	0.894 (0.450-1.779)	0.880 (0.442-1.754)	0.938 (0.469-1.876)
Model 3[c]	1	0.894 (0.450-1.779)	0.880 (0.442-1.754)	0.938 (0.469-1.876)
**Elevated waist circumference**				
Crude model	1	0.958 (0.477-1.926)	1.021 (0.506-2.059)	1.951 (0.906-4.202)
Model 1[a]	1	0.888 (0.430-1.833)	0.915 (0.442-1.893)	1.347 (0.599-3.028)
Model 2[b]	1	1.329 (0.452-3.907)	1.212 (0.416-3.526)	3.008 (0.835-10.841)

Model 3[c]	1	1.329 (0.452-3.907)	1.212 (0.416-3.526)	3.008 (0.835-10.841)
**Elevated percent body fat**				
Crude model	1	1.019 (0.472-2.200)	1.019 (0.472-2.200)	1.082 (0.497-2.359)
Model 1[a]	1	1.184 (0.510-2.751)	1.102 (0.475-2.558)	0.983 (0.401-2.409)
Model 2[b]	1	1.807 (0.513-6.369)	1.039 (0.297-3.640)	0.889 (0.217-3.640)
Model 3[c]	1	1.665 (0.459-6.037)	0.906 (0.242-3.391)	0.718 (0.153-3.378)

Abbreviations: MetS: Metabolic syndrome; GI: Glycemic index; OR: Odds ratio; CI: Confidence interval; HDL-C: High Density Lipoprotein-Cholesterol.
[a]Model 1: adjusted for age and gender.
[b]Model 2: adjusted for age, gender, BMI, smoking status, alcohol intake, energy intake, total fiber intake, sedentary behavior and education level.
[c]Model 3: adjusted for age, gender, BMI, smoking status, alcohol intake, energy intake, total fiber intake, sedentary behavior and education level and percentage of energy from protein and fat.

Significant results are shown in bold.

Table S5 Multivariable logistic regression analyses of MetS components and adiposity indicators by dietary GL 1 quartiles

	Daily Glycemic Load 1			
	**Quartile 1** (n=71)	**Quartile 2** (n=72)	**Quartile 3** (n=72)	**Quartile 4** (n=71)
	OR (95% CI)			
**Elevated triglycerides**				
Crude model	1	0.671 (0.311-1.447)	0.788 (0.372-1.669)	1.601 (0.790-3.245)
Model 1[a]	1	0.579 (0.263-1.276)	0.532 (0.237-1.192)	0.869 (0.389-1.938)
Model 2[b]	1	**0.425 (0.181-0.995)**	0.460 (0.198-1.067)	0.810 (0.351-1.871)
**Elevated fasting blood glucose**				
Crude model	1	1.233 (0.614-2.476)	1.091 (0.540-2.204)	1.212 (0.600-2.447)
Model 1[a]	1	1.159 (0.561-2.397)	0.763 (0.354-1.645)	0.974 (0.438-2.168)
Model 2[b]	1	1.025 (0.484-2.169)	0.755 (0.344-1.657)	0.973 (0.430-2.201)
**Elevated blood pressure**				
Crude model	1	1.488 (0.678-3.265)	1.460 (0.666-3.200)	**2.498 (1.173-5.320)**
Model 1[a]	1	1.285 (0.559-2.956)	0.779 (0.321-1.890)	1.392 (0.578-3.351)
Model 2[b]	1	1.116 (0.464-2.686)	0.773 (0.305-1.956)	1.441 (0.574-3.618)
**Reduced HDL-C[*]**				
Crude model	1	0.815 (0.411-1.617)	1.099 (0.561-2.152)	1.086 (0.552-2.138)
Model 1[a]	1	0.815 (0.411-1.617)	1.099 (0.561-2.152)	1.086 (0.552-2.138)
Model 2[b]	1	0.729 (0.360-1.477)	1.112 (0.563-2.195)	1.122 (0.565-2.226)
**Elevated waist circumference**				
Crude model	1	1.279 (0.595-2.747)	0.731 (0.356-1.499)	0.672 (0.328-1.376)
Model 1[a]	1	1.307 (0.594-2.877)	0.653 (0.309-1.380)	0.842 (0.398-1.779)
Model 2[b]	1	1.559 (0.481-5.058)	0.525 (0.187-1.477)	0.831 (0.278-2.486)

**Elevated percent body fat**				
Crude model	1	1.390 (0.566-3.414)	0.672 (0.301-1.502)	**0.388 (0.179-0.839)**
Model 1[a]	1	1.753 (0.677-4.540)	0.899 (0.373-2.168)	0.984 (0.393-2.460)
Model 2[b]	1	**4.472 (1.023-19.548)**	2.703 (0.590-12.389)	2.260 (0.345-14.806)

Abbreviations: MetS: Metabolic syndrome; GL: Glycemic load; OR: Odds ratio; CI: Confidence interval; HDL-C: High Density Lipoprotein-Cholesterol.
[a]Model 1: adjusted for age and gender.
[b]Model 2: adjusted for age, gender, BMI, smoking status, alcohol intake, energy intake, total fiber intake, sedentary behavior and education level.
Significant results are shown in bold.
